# Shugan Jieyu Capsule in Post-Stroke Depression Treatment: From Molecules to Systems

**DOI:** 10.3389/fphar.2022.821270

**Published:** 2022-01-24

**Authors:** Meng Zhang, Xue Bai

**Affiliations:** Department of Gerontology and Geriatrics, Shengjing Hospital of China Medical University, Shenyang, China

**Keywords:** Pharmacology, post-stroke depression, shugan jieyu capsule, traditional Chinese medicine, treatment

## Abstract

Post-stroke depression (PSD) is the most common non-cognitive neuropsychiatric complication after stroke, and about a third of patients with stroke have depression. Although a great deal of effort has been made to treat PSD, the efficacy thereof has not been satisfactory, due to the complex pathological mechanism underlying PSD. In Traditional Chinese Medicine (TCM) theory, PSD is considered to be a combination of “stroke” and “Yu Zheng*.*” The holistic, multi-drug, and multi-objective nature of TCM is consistent with the treatment concept of systems medicine for PSD. TCM has a very long history of being used to treat depression, and various TCM prescriptions have been clinically proven to be effective in improving depression. Among the numerous prescriptions for treating depression, Shugan Jieyu capsule (SG) is one of the classic prescriptions. Additionally, clinical studies have increasingly confirmed that using SG alone or in combination with Western medicine can significantly improve the psychiatric symptoms of PSD patients. Here, we reviewed the mechanism of antidepressant action of SG and its targets in PSD pathologic systems. This review provides further insights into the pharmacological mechanism, drug interaction, and clinical application of TCM prescriptions, as well as a basis for the development of new drugs to treat PSD.

## Introduction

Post-stroke depression (PSD) is a serious psychiatric condition that develops after stroke, and its prevalence is related to the time point after stroke. The cumulative incidence of PSD within 5 years after stroke is 39–52%, which usually occurs in the first month after stroke, and then gradually increases (Ayerbe et al., 2013).

The clinical manifestations of PSD are depression, guilt or low self-worth, sleep disturbances, fatigue, inattention, and suicidal tendencies (Das and Rajanikant, 2018). PSD has significant negative effects on physical, cognitive, and functional rehabilitation, and has become a serious human, social, and public health problem, reducing the survival rate and delaying the recovery of patients after stroke (Robinson and Jorge, 2016).

The pathophysiological mechanism of PSD is complex and multi-factorial (Ezema et al., 2019). Despite many clinical and basic studies, the pathophysiological mechanism of PSD remains incompletely elucidated. Currently, it is believed that the mechanism that may lead to PSD mainly include (i) dysregulation of the hypothalamic‒pituitary‒adrenal (HPA) axis (pro-inflammatory cytokines stimulate the HPA axis to release glucocorticoids associated with PSD) (Otte et al., 2016; Robinson and Jorge, 2016); (ii) glutamate-mediated toxicity mechanism (in stroke patients, a higher serum glutamate level may be involved in PSD through cerebral infarction formation) (Cheng et al., 2014; Gruenbaum et al., 2020); (iii) monoamine transmissions (PSD may be associated with lower levels of biological monoamines, i.e., serotonin [5-HT], norepinephrine [NE], and dopamine [DA], in the central nervous system [CNS]); (iv) neurotrophic factor dysregulation (brain-derived neurotrophic factor [BDNF]-deficiency contributes to the pathophysiology of PSD) (Zhang and Liao, 2020); (v) increased inflammatory cytokine levels (interleukin [IL]-1, IL-6, and interferon γ [IFN-γ]) are important factors leading to PSD (Li J. et al., 2014). In addition, intestinal microflora disorder, serotonin transporter (5-HTT) gene polymorphism, and mitochondrial dysfunction may also be involved in the pathophysiology of PSD (Jiang et al., 2015; Bansal and Kuhad, 2016).

Clinically commonly used anti-PSD drugs include tricyclic antidepressants, monoamine oxidase inhibitors, selective 5-HT-reuptake inhibitors, 5-HT‒NE reuptake inhibitors, and specific serotonergic antidepressants. However, these CNS-orientated, single-target, and conventional antidepressants are insufficient and far from ideal for PSD therapy (Tao et al., 2021). On the other hand, traditional Chinese medicine (TCM) has been used to treat depression for thousands of years. The holistic, multi-drug, and multi-objective nature of TCM is highly consistent with the treatment concept of systems medicine in PSD.

In our mini-review, we will explain the pathogenesis of PSD from the perspective of TCM theory, briefly review the TCM prescriptions for the treatment of PSD, introduce in detail the composition of the classic PSD prescription Shugan Jieyu capsule (SG) and its active ingredients, and consider the pathology of PSD, in order to provide a deeper understanding of the pharmacological mechanism, drug interaction, and clinical application of TCM prescriptions.

## PSD According to TCM

PSD is a comorbidity of “stroke” and “Yu Zheng”. Based on the theory of “the identity of the collateral and vascular system,” it is concluded that cerebral collateral stasis (blood collateral stasis)/cerebral vascular occlusion-caused brain tissue loss is the key pathogenesis of stroke. Qi collaterals (络) are related to the neuroendocrine functions in modern medicine, and neuroendocrine functions are associated with the occurrence of depression. Therefore, it is speculated that there may also be correlation between qi collaterals and Yu Zheng. In the meridians, the “meridian qi” of the circulation around the body is disseminated to the whole body through qi collateral channels, which play the roles of nourishing the viscera, transmitting information, defense and protection, and regulating and controlling the function of the whole body.

The “meridian qi circulation system” is quite consistent with the neuroendocrine regulation system of Western medicine (Loubinoux et al., 2012). Yu Zheng patients have suffered an emotional injury, resulting in abnormal changes in qi, leading to stagnation of collateral qi, and inhibition of blood flow. Qi and blood deficiency of collaterals and loss of nourishment via brain collaterals result in emotional depression, lack of interest and/or pleasure, and other mental symptoms. PSD, as a “stroke” and “Yu Zheng” comorbidity, involves qi and collateral disorders, and blood and collateral obstruction. The main characteristics of the treatment of collateral diseases is “Activating meridians”, among which regulating the Zang-Fu organs is the fundamental treatment.

PSD is caused by the interaction of liver qi stagnation and blood stasis after stroke. On the basis of liver qi stagnation, PSD may combine with heart, spleen, kidney deficiency and phlegm stasis. Some scholars also believe that PSD is depression caused by stroke, which is based on deficiency of kidney essence, deficiency of qi, blood and Yin and Yang of Zang-Fu organs, and marked by qi stagnation, blood stasis and phlegm turbidity. The pathogenesis is liver qi stagnation, and the key is spleen failure. The pathogenesis can be transformed into each other, and is mixed with deficiency and reality.

## TCM in PSD Treatment

The application of classical prescriptions of TCM to treat diseases has a long history. Ancient literature contains many therapeutic prescriptions of Yu Zheng. Modern doctors and scholars use classical prescriptions to treat PSD, and continue to perform clinical and scientific research to provide a basis for the development of syndrome differentiation and treatment by classical prescriptions.

Chaihu Shugan San (CSS) is a classic and effective antidepressant TCM that has been used in China for thousands of years. CSS plays an antidepressant role by regulating the BDNF/extracellular signal-regulated kinase (ERK)/cAMP-response element binding protein (CREB) signaling pathway in the hippocampus and frontal cortex (Yan et al., 2020). Buyang Huanwu decoction ameliorates PSD by promoting neurotrophic pathway-mediated neuroprotection and neurogenesis (Luo et al., 2017). Xiaoyao formula (XYF) is a well-known Chinese herbal formula for treating “Liver Stagnation and Spleen Deficiency” syndrome. The beneficial effects of XYF on PSD with few adverse events have been shown by studies in China (Jin et al., 2018). Huanglian-Wendan decoction (HWD) has antidepressant activity with hepatoprotection in rat models of chronic, unpredictable, mild stress, which was associated with its anti-inflammation action both peripherally and centrally. The inhibitory modulation of NF-κB and nucleotide binding oligomerization domain 3-like receptor (NLRP3) inflammasome activation by HWD may mediate its antidepressant action (Jia et al., 2018). Chaihu Jia Longgu Muli decoction (CLMD) is widely used in the treatment of PSD in China. Current evidence suggests that CLMD is more effective and safer for the treatment of PSD than Western oral antidepressants (Wan et al., 2021). CLMD can regulate HPA axis dysfunction by preventing dopaminergic and serotonergic transmission in the prefrontal cortex (Wan et al., 2021) and upregulating BDNF expression to alleviate the depression-like state induced by chronic stress (Chen et al., 2020). Tongqiao Huoxue decoction (TQHXD) exerts an apparent protective effect on glutamate-damaged neurocytes in terms of proliferation activity and membrane permeability, which shows its potential to treat PSD (Wang et al., 2010). As the expression levels of BDNF is decreased in PSD, Yinao Jieyu recipe could inhibit the progress of PSD (Li et al., 2015a).

The results of a clinical controlled study in China suggested that Jieyu Huoxue Decoction can not only relieve PSD, but also improve neurological impairment (Feng et al., 2004). Xingnao Jieyu (XNJY) decoction can treat PSD through multiple mechanisms (Fan et al., 2012). For example, XNJY promotes BDNF expression by regulating the BDNF/ERK/CREB signaling pathway (Li et al., 2018). In addition, XNJY upregulates synaptotagmin expression in hippocampi and exerts antidepressant effects (Yan et al., 2013). Peng et al. suggested that Wuling capsules have a certain antidepressant effect, and can be used as a treatment option for PSD (Peng et al., 2014).

SG is a TCM that is used to improve cognitive impairment and emotional disorders caused by PSD (Yao et al., 2020). Emerging studies have demonstrated that SG alone or combined with Western antidepressants has a satisfactory therapeutic effect on PSD, with no obvious adverse reactions. Below, we will elaborate on the active constituents of SG and the pharmacological mechanism of SG in treating PSD.

## Molecular Mechanism of Action of SG Antidepressants

SG is mainly composed of *Hypericum perforatum* and *Acanthopanax*. *Hypericum perforatum* has the functions of clearing heat and detoxifying, soothing the mind, cooling the blood, and nourishing Yin. It has a long history of use in treating mental diseases and has been recognized in the modern Chinese medicine circle. *Acanthopanax* has the effect of replenishing qi and invigorating the brain. It is commonly used to treat depression, neurosis, and neurasthenia. In the treatment process, these two compounds cooperate to produce a calming and tranquilizing anti-depressant effect.

*Hypericum perforatum* is a perennial herb with erect stems and many branches, axillary branches, dense opposite leaves that are elliptic to linear, 1–2-cm long, and 3–9-mm wide. Flowers are born at the stem apex or branch tip, with cymes, five lanceolate sepals, and black glandular spots at the margin. Its taste is spicy and slightly bitter. The whole plant contains phenylpropane, flavonol derivatives, flavonoids (diflavones), proanthocyanidins, oxyxanthanone, phloroglactil (hypericin and its homologue pseudohypericin), hypericin and its derivatives, and some precursors (prohypericin, hypericin auxiliary dehydrodione). The content of hypericin is lower in young plants, higher in the flowering period, and lowest in the stems. It also contains anthraphenol compounds, such as emodin anthraphenol. Its flavonoid content is 3.35–7.4%, mainly hyperoside and rutin, but also quercetin, isoquercitrin, and miquelianin. In addition, a new antibacterial substance, novlimanin, which is a bicyclic tetraketone compound containing four isopentene chains, was isolated from an acetone extract of the plant (Cirak et al., 2010; Verjee et al., 2019).

*Acanthopanax* is a deciduous shrub that grows to 2 m tall and has stems that are usually dense, with slender barbs. Its medicinal ingredients mainly come from its roots, rhizomes, or stems and leaves. The rhizome is irregularly cylindrical, 1.4–4.2 cm in diameter. The root is cylindrical, 0.3–1.5 cm in diameter. It is slightly fragrant and tastes mildly spicy and bitter. Modern pharmacological studies have found that *Acanthopanax* contains glycosides, polysaccharide, fatty acids, quinones, amino acids and trace elements. The main glycosides are *Acanthopanax* glycosides A, B, C, D, E, F, and G. Polysaccharides mainly include glucose, sucrose, alkali-soluble polysaccharide and water-soluble polysaccharide. Fatty acids and quinones mainly include methyl oleate, ethyl oleate, 10, -octadecadienoic acid, 10, -octadecadienoic acid, ethyl ester, mymyritic acid, palmitic acid, 9, 11-octadecadienoic acid, hexadecadienoic acid (Li et al., 2021).

**FIGURE 1:**
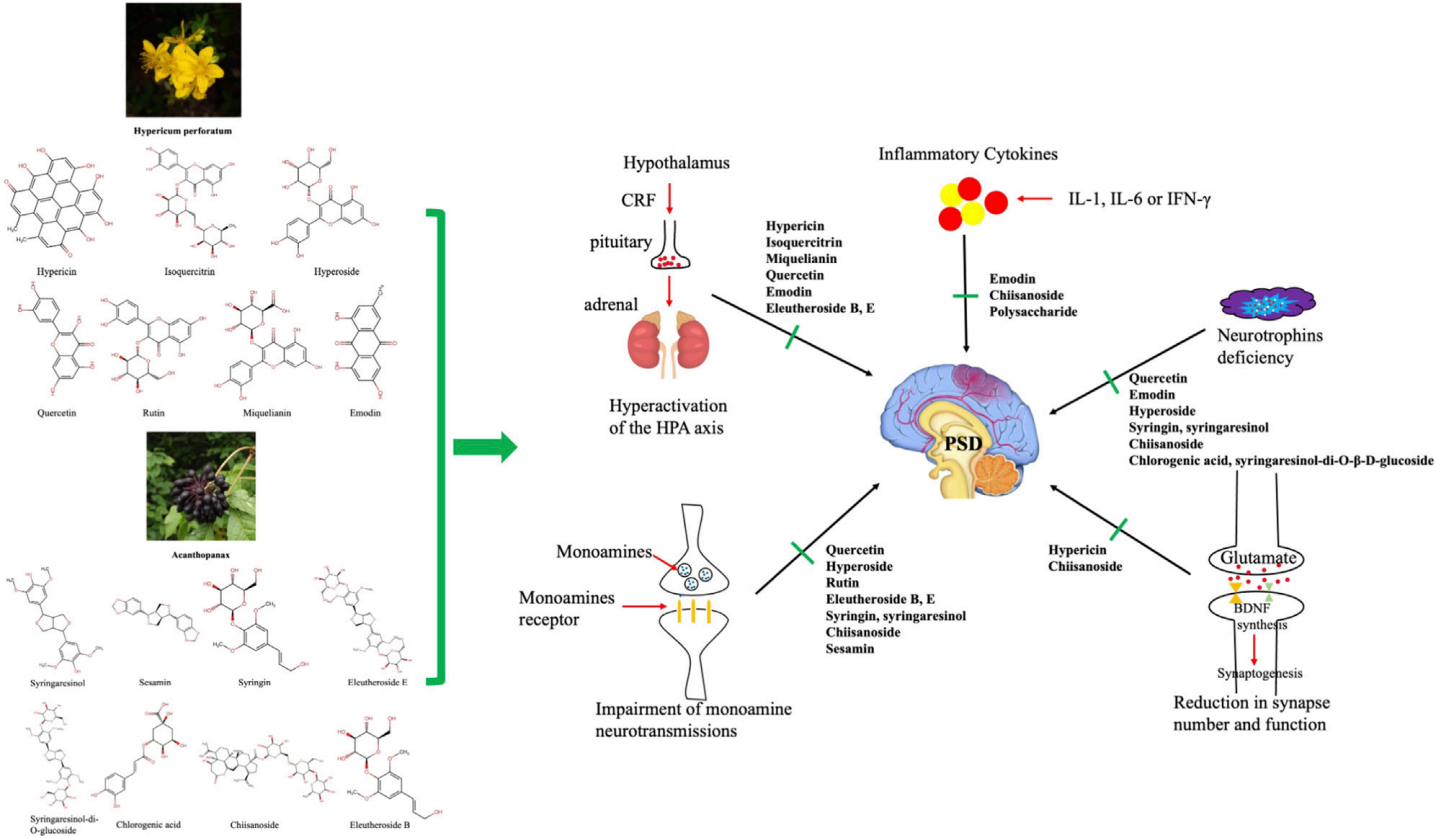
Several divergent systems are involved in the pathophysiology of post-stroke depression. The active ingredients (the 2D structures on the left) in Shugan Jieyu capsules may act on the pathophysiological system of the central nervous system, including the hypothalamus‒pituitary‒adrenal axis, monoamine neurotransmission, neurotrophins, inflammatory factor release, and synapse number and function as shown.

Through a literature review, we found that a variety of chemical components of the two TCMs may play a role in the treatment of PSD by regulating the HPA axis, glutamate transmission, monoamine transmission, BDNF, and other mechanisms (Table 1; Figure 1). Next, we explore the pharmacological mechanisms of the chemical components of these two compounds at the molecular level, from several pathogenically mechanisms involved in PSD.

**TABLE 1:** Herbal constituents in Shugan Jieyu Capsule (SG) that produce antidepressant-like activities in animal models or cells.

SC composition	Herbal constituents	Mechanism of action	Models	Administration dosage	Treatment time	Reference
*Hypericum perforatum*	Hypericin	HPA axis/Glutamate transmission	Male CD rats	0.2 mg/kg	8 weeks	Butterweck et al. (2001)
	Isoquercitrin	HPA axis	Male CD rats	0.6 mg/kg	2 weeks	Butterweck et al. (2004)
	Miquelianin	HPA axis	Male CD rats	0.6 mg/kg	2 weeks	Butterweck et al. (2004)
	Quercetin	Monoamine transmissions/HPA/BDNF	Wistar rats	10 mg/kg	60 min	Herraiz and Guillén, 2018; Kawabata et al., 2010
	Emodin	BDNF/the HPA axis	CUMS rats	40 or 80 mg/kg	6 weeks	Li et al., 2014b
		Anti-inflammatory response	CUMS rats	80 mg/kg	2 weeks	Zhang et al. (2021)
	Hyperoside	BDNF	Male SD rats	10 mg/kg	34 days	Gong et al. (2017)
		Monoamine transmissions	CUMS rats	3.75 mg/kg	24 h	Orzelska-Górka et al. (2019)
	Rutin	Monoamine transmissions	Male Swiss mice	3.0 mg/kg	4 days	Machado et al., 2008
*Acanthopanax*	Eleutheroside B, E	HPA axis	Male ICR mice	-	14 days	Jung et al. (2013)
		Monoamine transmissions	Male Kunming mice	15 mg/kg	21 days	Qi et al. (2020)
	Syringin, syringaresinol	Monoamine transmissions	Male Kunming mice	−	7 days	Jin et al. (2013)
		BDNF	PC12 cell	−	12 h	Wu et al. (2013)
	Chiisanoside	Monoamine transmissions/Anti-inflammatory response/BDNF/Glutamate transmission	Male ICR mice	5.0 mg/kg	7 days	Bian et al. (2018)
	Polysaccharide	Anti-inflammatory response	Male Kunming mice	300 mg/kg	14 days	Han et al., 2016
	Chlorogenic acid, syringaresinol-di-O-β-D-glucoside	BDNF	Male Sprague Dawley rats	40 mg/kg and 32 mg/kg	7 days	Miyazaki et al. (2020)
	Sesamin	Monoamine transmissions	Male rats of the Lewis strain	30 mg/kg	14 days	Fujikawa et al., 2005

### HPA Axis

There is a neuro-hormone‒inflammatory interaction between the HPA axis and the CNS, endocrine system, or immune system, which forms an integrated network at the basis of PSD pathogenesis. Stress leads to activation of the HPA axis, which is typically manifested by high levels of glucocorticoids, leading to depressive symptoms by impairing neuronal survival and neurogenesis (Keller et al., 2017). Abnormal inflammatory responses and higher glucocorticoid levels are associated with PSD, and increased inflammation and dysregulation of the HPA may increase the risk of PSD through multiple mechanisms (Villa et al., 2018; Wen et al., 2018). Monoamine antidepressants not only reverse stress-induced hyperactivity of the HPA axis, but also attenuate the inflammatory response by reducing the release of pro-inflammatory cytokines in activated microglia (Simões et al., 2019). Similarly, agents that eliminate inflammatory effects may exert antidepressant activity in animal models through the interaction between the immune system and the CNS (Zunszain et al., 2011). In addition, antidepressant agents directly acting on the HPA axis, such as glucocorticoid receptor antagonists, can also be effective as antidepressants by blocking the receptor activity and terminating the hormone over-secretion caused by stress-induced HPA axis hyperactivity (Menke, 2019).

The effect of hypericin (the active component of *Hypericum perforatum*) on gene expression may be involved in the regulation of the HPA axis. Butterweck et al. demonstrated that, after 8 weeks of intragastric administration of hypericin (0.2 mg/kg), corticotropin-releasing hormone and 5-HT receptor 1A receptor mRNA was significantly reduced in rats’ hypothalamic paraventricular nucleus (Butterweck et al., 2001).

Flavonoids (isoquercitrin and miquelianin) are also active constituents of *Hypericum perforatum* that modulate HPA axis function. Isoquercitrin and miquelianin given daily by gavage for 2 weeks significantly down-regulated circulating plasma levels of adreno-corticotropic hormone (ACTH) and corticosterone in rats (Butterweck et al., 2004).

Eleutheroside B and E are ethanol extracts of *Acanthopanax*. In mice with stress-induced behavioral alterations, administration of eleutheroside B and E could potently activate the HPA axis to decrease the elevated corticosterone concentration and relieve major depressive disorder (Jung et al., 2013). Qi et al. (2020) also drew a similar conclusion in depression-model mice: eleutheroside could significantly shorten the tail suspension test, and improve the contents of DA, NE, and 5-HT in the brain of mice.

### Glutamate Transmission

Changes in central glutamate neurotransmission are related to the pathophysiology of depression and the mechanism of action of antidepressants (Duman et al., 2019). In stroke patients, the correlation between higher serum glutamate levels, infarct volume, and depression suggests that glutamate may be involved in PSD through stroke formation (Chen, 2014). N-methyl-D-aspartic acid receptor channel blockers and acetylcholine muscarinic receptor antagonists enhance glutamate transmission, and then increase BDNF release and synaptic function, so as to reverse stress-induced synaptic abnormalities rapidly (Burgdorf et al., 2013). Among them, ketamine, as an NMDA receptor antagonist, has been proven to produce rapid antidepressant effects (Murrough et al., 2013).

Inhibition of glutamate release is also one of the potential mechanisms of hypericin’s antidepressant effects. Hypericin has been reported to inhibit neuroglutamate release in the rat cortex by reducing voltage-dependent Ca^2+^ influx and mitogen-activated protein kinase activity (Chang and Wang, 2010).

Chiisanoside, a triterpenoid saponin extracted from *Acanthopanax*, may have antidepressant activity (Bian et al., 2018). In depression-model mice, it has been suggested that chiisanoside effectively increases the DA and γ-aminobutyric acid levels. Chiisanoside administration could also reduce serum IL-6 and tumor necrosis factor-α (TNF-α) levels. Moreover, the changes in oxidative stress-related indicators were effectively improved. Additionally, chiisanoside inhibited TrkB, BDNF, and NF-κB in the hippocampus (Bian et al., 2018). Therefore, chiisanoside exhibited significant antidepressant-like effects via multiple pharmacological mechanisms.

### Monoamine Transmissions

Monoamine neurotransmission injury may be involved in the pathological mechanism of PSD. Ischemic injury of amine-containing axons from the brainstem to the left cerebral cortex may lead to decreased 5-HT and NE synthesis in the frontal, temporal, and basal ganglia limbic regions, leading to PSD. Monoamine-reuptake transporters of 5-HT and NE are the main targets of antidepressant drugs. Monoamine-based inhibitors enhance 5-HT or NE transmission by different mechanisms, leading to altered discharge activity in the dorsal raphe nucleus or locus coeruleus, thereby improving PSD (Araragi et al., 2013).

Quercetin is one of the flavonoids in *Hypericum perforatum*. Herraiz et al. reported that quercetin and its glycosides can inhibit the activity of monoamine oxidase A, and could be a potential antidepressant for treating PSD through its antioxidant activity (Herraiz and Guillén, 2018). Quercetin also significantly inhibited the increase of plasma corticosterone and adrenocorticotropic hormone levels by inhibiting the expression of corticotropin-releasing factor mRNA (Kawabata et al., 2010). In a focal cerebral ischemia model, quercetin could activate the BDNF‒TrkB‒PI3K/Akt signaling pathway. Therefore, quercetin’s antidepressant-like activity is related to its ability to modulate levels of BDNF (Yoshino et al., 2011).

The antidepressant activity of hyperoside was evaluated by Orzelska-Górka et al. They demonstrated that hyperoside was able to reverse depressive symptoms in the forced swimming test and sucrose-preference test in rats exposed to chronic mild stress. Further pharmacological studies suggested that this antidepressant effect may be mediated in part by regulation of the central monoamine neurotransmitter system and increased levels of BDNF (Cai et al., 2017). This result may facilitate further study of the antidepressant mechanism of this potential treatment option (Orzelska-Górka et al., 2019).

Rutin, a diglycoside flavonol, is extensively found in plants, including *Hypericum perforatum*. It has therapeutic potential for depression: on the one hand, rutin prevented the overexpression of inflammatory biomarkers, such as cyclooxygenase-2, IL-8, and inducible nitric oxide synthase, while on the other hand, it increased the availability of 5-HT, NE, and DA in the synaptic cleft (Pathak et al., 2013; Souza et al., 2018; Liu et al., 2021).

Syringin and syringaresinol are the main active ingredients of *Acanthopanax*. Jin et al. suggested that syringin and syringaresinol could significantly elevate the levels of 5-HT, NE, and DA in the whole brain of mice. In addition, levels of CREB protein expression were also upregulated by syringin and syringaresinol pre-treatment of depression-model mice. Therefore, the antidepressant mechanism of syringin and syringaresinol may be mediated *via* the central monoaminergic neurotransmitter system and CREB protein expression. Administration of syringin and syringaresinol may be beneficial for patients with depressive disorders (Jin et al., 2013). In terms of corticosterone-induced neurotoxicity in PC12 cells, syringin and syringaresinol could increase BDNF mRNA levels and CREB protein expression, and increased PC12 cell viability, which may be mechanisms accounting for the *in vivo* antidepressant activity of *Acanthopanax* (Wu et al., 2013).

### Neurotrophins

BDNF is the most abundant and widely distributed neurotrophic factor in the CNS. It plays a crucial role in the growth and differentiation of the nervous system. The level of BDNF decreased in both the brain of patients with depression and in animal models of depression (Luo et al., 2013). In addition, increasing clinical and experimental evidence has shown that changes in BDNF levels are associated with beneficial therapeutic activities of antidepressants (Thompson Ray et al., 2011). Therefore, BDNF is considered a possible target for antidepressants.

Emodin is one of the active chemical constituents of *Hypericum perforatum*. Previous studies have shown that emodin can protect the brain from the excitatory toxicity of glutamate by reducing its release (Bonaterra et al., 2020). Emodin has recently been reported to oppose chronic, unpredictable, mild stress-induced depressive-like behavior in mice by upregulating the levels of hippocampal glucocorticoid receptor and BDNF (Li M. et al., 2014).

In addition, emodin also had anti-inflammatory activities: it prevented depressive behaviors in DeS rats by inhibiting microglial activation, down-regulating IL-1β, TNF-α, and 5-lipoxygenase expression (Wang et al., 2018). Moreover. Zhang et al. demonstrated that emodin inhibited excess inflammatory response by targeting miR-139-5p/5-lipoxygenase (Zhang et al., 2021). Hyperoside increased the expression of BDNF in the hippocampus of CUMS rats, but it did not affect the level of plasma corticosterone (CORT), so it may be one of the potential reasons for the antidepressant effect of *Hypericum perforatum* (Gong et al., 2017).

Recent studies have identified chlorogenic acid and syringaresinol-di-O-β-D-glucoside (SYG) as bioactive components of *Acanthopanax*. Chlorogenic acid and SYG increases hippocampal BDNF protein levels, activates hippocampal BDNF signaling, therefore, the anti-depressive effects of *Acanthopanax* may be through the regulation of BDNF expression and its downstream signaling (Miyazaki et al., 2020).

Sesamin is an active integrant of *Acanthopanax* and has been identified as a new anti-metalloproteinases inhibitor (Załuski et al., 2016). In rotenone-induced Parkinsonian rats, Baluchnejadmojarad et al. demonstrated that sesamin could provide cytoprotective effects on DA cells in the substantia nigra and relieve depression in Parkinson disease (Baluchnejadmojarad et al., 2017).

### Inflammatory Cytokines

A large number of studies have shown that cytokines are involved in both the inflammatory response of acute ischemic stroke and in depression, and thus may play a role in the occurrence of PSD. Inflammatory factors, such as IL-1, IL-6, and IFN-γ are important factors leading to postmenopausal depression (Wang et al., 2013). In addition, other inflammatory factors, such as TNF-α, IL-8, IL-18, and hypersensitive-C-reactive protein have also been found to play an important role in the pathogenesis of PSD (Cheng et al., 2018; Wu et al., 2020).

Polysaccharides, derived as the aqueous extract of *Acanthopanax,* are its major active ingredient. Previous studies have shown that polysaccharides have immune regulatory activity and inflammation inhibitory activity in animal models (Chen et al., 2011). Polysaccharides could decrease lipopolysaccharide-induced secretion of inflammatory mediators, including TNF-α and NF-κB (Han et al., 2014), demonstrating the potential of polysaccharides to treat PSD through anti-inflammatory actions.

## Clinical Application of SG in the Treatment of PSD

**FIGURE 2:**
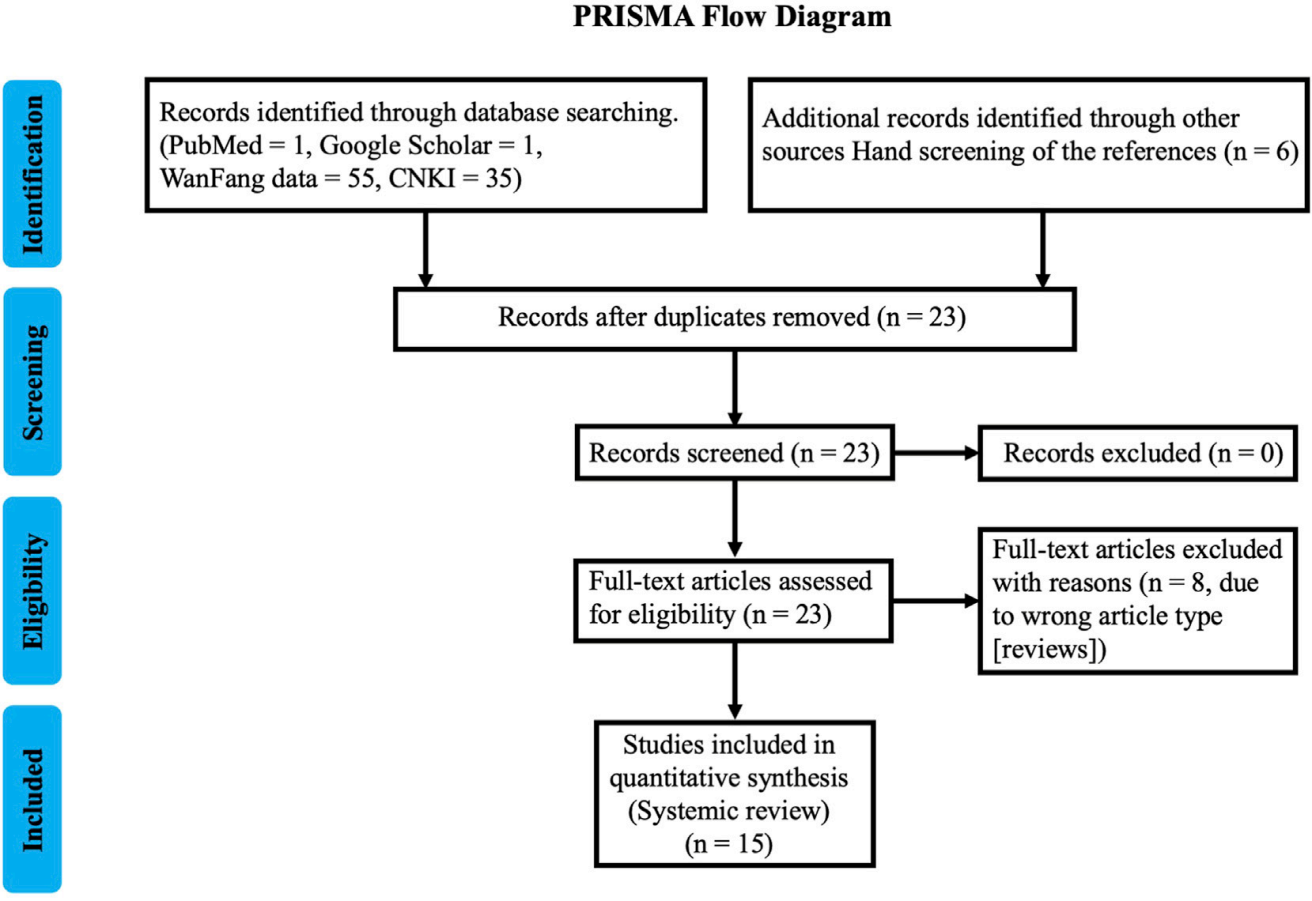
Prisma flowchart with details of the article screening process.

A systemic literature search was performed for articles using PubMed, Web of Science, Cochrane, CNKI (https://www.cnki.net/), Weipu (http://www.cqvip.com/), and Wanfang (http://www.wanfangdata.com.cn/index.html) database for the period 1990–2021. The search term “Shugan Jieyu Capsule” and “Post-Stroke Depression” were used. The quality of the added cases was assessed by two reviewers independently (ZB and XD) using the Joanna Briggs Institute case report appraisal checklist for inclusion in systematic reviews (Munn et al., 2020). We found 809 case of PSD in 15 publications (Figure 2; Table 2). All studies suggested SG have obvious therapeutic effect on PSD patients. In addition, much evidence has confirmed the efficacy of SG combined with Western medicine in the treatment of PSD (Table 3). Zeng et al. suggested that PSD treatment with SG for 6 weeks had a higher overall effectivity rate than paroxetine (78.0 *vs*. 56.2%) and a lower incidence of side effects (7.3 *vs*. 24.4%) (Zeng et al., 2014). In addition, SG can be combined with a variety of classic Western antidepressant drugs to treat PSD and has achieved an ideal therapeutic effect. For example, SG can be combined with venlafaxine, paroxetine, and citalopram. Compared with PSD patients using Western drugs alone, SG showed better efficacy and provided patients with a safer treatment experience (Zhang and Xu, 2013; Zheng et al., 2014). Moreover, compared with agomelatine alone, SG combined with agomelatine significantly improved the metabolic levels of neurocytokines and monoamine transmitters in PSD patients, further proving the effectiveness of SG from a pathogenesis perspective (Fu and Wang, 2019).

**TABLE 2:** Characteristics and findings in clinical studies reviewed.

Authors, years	Study type	Country location	Study group	Age (years); sex	Therapeutic intervention	Treatment course	Main efficacy evaluation index	Outcome
Wu et al. (2014)	RS	China	30 P	55.2 ± 9.3; 17 male, 13 female	SG 720mg, Bid	6 weeks	TER, HAMD	Improve
Pang, (2019)	RS	China	50 P	68.1 ± 5.5; 32 male, 18 female	SG 720mg, Bid	4 weeks	SDS	Improve
Zeng et al. (2014)	RS	China	80 P	62.3 ± 9.2; 44 male, 38 female	SG 720mg, Bid	6 weeks	SDS, ADR	Improve
Li et al. (2015b)	RS	China	157 P	32–52, 75 male, 82 female	SG 720mg, Bid	6 weeks	HAMD	Improve
Cheng, (2012)	RS	China	50 P	52.0 ± 8.0; 29 male, 21 female	SG 720mg, Bid	6 weeks	TER, HAMD	Improve
Xiong and Li, (2015)	RS	China	30 P	46.3 ± 5.2; 18 male, 12 female	SG 720mg, Bid	6 weeks	NIHSS, BI, HAMD	Improve
Li, 2019slu	RS	China	41 P	67.2 ± 5.8; 22 male, 19 female	SG 720mg, Bid	6 weeks	HAMD	Improve
Zhao and Wu, (2013)	RS	China	64 P	42–77; 29 male, 35 female	SG 720mg, Bid	6 weeks	HAMD	Improve
Chen, (2014)	RS	China	58 P	58.5 ± 5.7; 29 male, 28 female	SG 720mg, Bid	6 weeks	HAMD	Improve
Lu and Gu, (2019)	RS	China	41 P	67.2 ± 5.8; 22 male, 19 female	SG 720mg, Bid	6 weeks	HAMD	Improve
Liu et al., 2019	RS	China	59 P	67.2 ± 5.8; 35 male, 19 female	SG 720mg, Bid	4 weeks	SDS, HAMD, ADL, NFDS	Improve
Zheng et al. (2017)	RS	China	40 P	59.3 ± 4.0; 23 male, 17 female	SG 720mg, Bid	12 weeks	BI, HAMD	Improve
Yao et al. (2020)	RS	China	15 P	64.1 ± 6.0; 8 male, 7 female	SG 720mg, Bid	8 weeks	HAMD	Improve
Wang et al. (2016)	RS	China	34 P	66.5 ± 4.9; 18 male, 16 female	SG 720mg, Bid	6 weeks	HAMD	Improve
Mao and Luo, (2016)	RS	China	60 P	62.5 ± 3.8; 32 male, 28 female	SG 720mg, Bid	8 weeks	HAMD	Improve

ADL, activity of daily living scale; ADR, adverse drug reactions; BI, Barthel Index; HAMD, Hamilton Depression Scale; NFDS, neural function defect score; NIHSS, National Institute of Health stroke scale; P, patients; TER, total effective rate; RS, retrospective study; SDS, Self-rating depression scale; SG, Shugan Jieyu capsule.

**TABLE 3:** Characteristics and Findings in efficacy of SG combined with Western medicine in the treatment of PSD.

Number of patients	Degree of depression		Therapeutic intervention		Treatment course	Main efficacy evaluation index	Key findings	References
			**Treatment group**	**Control group**				
P 41: C 41		Mild-moderate	SG	Paroxetine	6 weeks	SDS	SG could effectively improve depression symptoms in PSD patients with high security	Zeng et al. (2014)
P 35: C 35		Moderate-severe	SG + Venlafaxine	Venlafaxine	6 weeks	HAMD	Venlafaxine combined with SC is effective and safe in the treatment of moderate to severe PSD	Zhang and Xu, 2013
P 32: C 32		Mild-moderate	SG + Paroxetine	Paroxetine	6 weeks	HAMD	SG combined with Paroxetine in treatment of PSD clinical efficacy with fewer adverse reactions	Zheng et al. (2014)
P 47: C 47		Mild-moderate	SG + Agomelatine	Agomelatine	6 weeks	HAMD	SG combined with agomelatine can improve the levels of neurocytokines and monoamine transmitter metabolism in PSD patients	Fu and Wang, (2019)
P 30: C 30		Mild-moderate	SG + Citalopram	Citalopram	6 weeks	HAMD, NIHSS	Citalopram combined with SG is effective and safe in the treatment of PSD	Xiong and Li, (2015)
P 48: C 48		Mild-moderate	SG + Venlafaxine	Venlafaxine	6 weeks	HAMD, NIHSS	SG increases neurotransmitter transmission, alleviates negative emotions and promotes the recovery of neurological function, with high safety	Wang et al. (2021)
P 30: C 21		Mild-moderate	SG	−	8 weeks	MoCA, HAMD	SG may improve the cognitive function of PSD patients through alteration of brain dynamics	Yao et al. (2020)
P 40: C 40		Mild-moderate	SG	−	6–12 weeks	HAMD, Barthel	SG therapy for PSD is effective and also improve the ability of daily life	Zheng et al. (2017)

Degree of depression: mild-moderate: 17 ≤ HAMD^17^ ≤ 28; moderate-severe 24 ≤ HAMD^17^.

HAMD, HAMD, Hamilton Depression Scale; MoCA, Montreal Cognitive Assessment; NIHSS, National Institutes of Health stroke scale; PSD, post-stroke depression; SDS, self-rating depression scale; P, patients; C, controls.

For PSD patients with motor symptoms, SG also showed advantages. SG increased neurotransmission, alleviated negative emotions, and promoted the recovery of neurological function, with high safety (Wang et al., 2021). Zheng et al. also concluded that SG therapy for PSD is effective and improved the abilities of daily living (Zheng et al., 2017). PSD may be accompanied by dynamic changes in brain function, thereby impairing the cognitive function of patients. The research results of Yao et al. suggested that SG may improve the cognitive function of PSD patients by changing brain dynamics, which lays a foundation for exploring the neurobiological mechanism of SG by improving the symptoms of PSD patients (Yao et al., 2020).

In summary, these clinical studies have shown that SG has obvious advantages in the treatment of PSD. In addition to improving the depressive symptoms of PSD patients, SG can also alleviate the physical dysfunction of PSD patients, increase their quality of life, and possibly improve their cognitive function. However, most of the current studies on the treatment of PSD by SG are limited to Chinese patients, and the number of patients included in the studies is small. Future large-scale randomized controlled studies are needed to confirm the efficacy of SG against PSD.

## Discussion

Using modern pharmacological research methods to explore the molecular mechanism of TCM components or single molecules can facilitate an understanding of the systematic mechanism behind TCM prescriptions. Systems pharmacology studies the effective components, drug targets, and effects of TCM at the systemic level, revealing all the responses of different biological systems to the pharmacological effects of the drugs (Li and Zhang, 2013). Therefore, systems pharmacology is an important approach for gaining understanding of the systemic mechanisms of TCM prescriptions. In addition, with the development of TCM prescription research, increasing numbers of TCM monomers have been discovered. Through *in vivo*, *in vitro*, animal, and human pharmacology studies, it has been found that there are many effective components in TCM for treating diseases, and these active components may play a synergistic role through various mechanisms.

Although single TCM molecules have been shown to have powerful effects in the treatment of PSD, compound TCM prescriptions, rather than single forms, are used clinically. In clinical practice, TCM prescriptions are more effective and safer than single drugs, probably due to their synergistic and mutual detoxification. SG is a TCM prescription that can be explained by systems pharmacological studies of the interactions between TCM molecules that may be triggered by the synergistic effects of two or more herbs.

However, research into the pharmacological mechanism of TCM prescriptions is still in its infancy, and the ratio and molecular interaction of various drugs in the prescriptions have not been clearly elucidated. At present, studies on pharmacological mechanisms can only prove the effectiveness of the active ingredients in TCM, and the synergistic effects of various active ingredients need to be explored further and confirmed. The effective way to solve this problem is to establish a complete database (Xie et al., 2015), including the pharmacological action of various drug monomers, the synergistic action of various monomers, and their interaction with various diseases. Such a database will facilitate advancement of research from the molecular level to the system level, and thereby promote the TCM treatment of PSD.

In conclusion, the combination of system level and molecular level research not only represents a trend in TCM research, but also promotes our understanding of the mechanism of the TCM prescription system. With the development of neurobiology and systems pharmacology, research into SG will help to develop appropriate drugs or methods for the effective and safe treatment of PSD in future.
